# Supplementary material for: AgNO3 Sterilizes Grains of Barley (Hordeum vulgare) without Inhibiting Germination—A Necessary Tool for Plant–Microbiome Research
Source: Plants (Basel). 2020 Mar 17;9(3):372. doi: 10.3390/plants9030372 (PMC7154866; doi:10.3390/plants9030372)
Supplement: Supplementary file 1 [file plants-09-00372-s001.pdf]

Supplementary Material

1 Supplementary Figures and Tables

1.1 Supplementary Figures

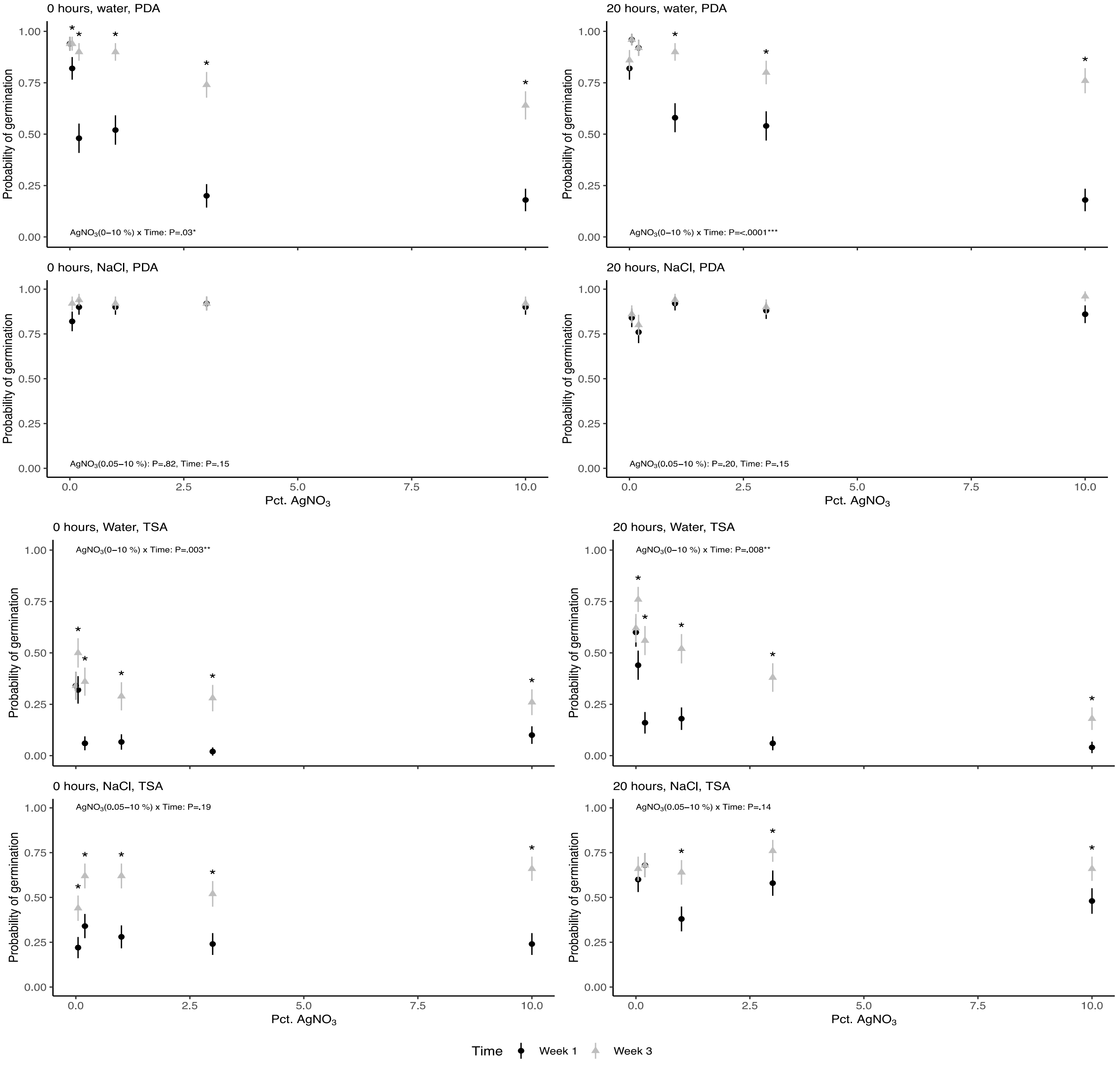

Figure S1. Difference in germination of grains in response to concentration of $AgNO_3$ between day 7 and 21. Symbols and error bars are mean probability ±SE of observed values (no. observations = 50 except 1% $AgNO_3$, dry grains rinsed in ddH_2O on TSA plates = 45). Asterisk indicates a significant difference (p-value<0.05) between day 7 (black circles) and day 21 (gray triangles) at that particular $AgNO_3$ concentration. Means and Tukey's test results are specified in detail in Table S3.

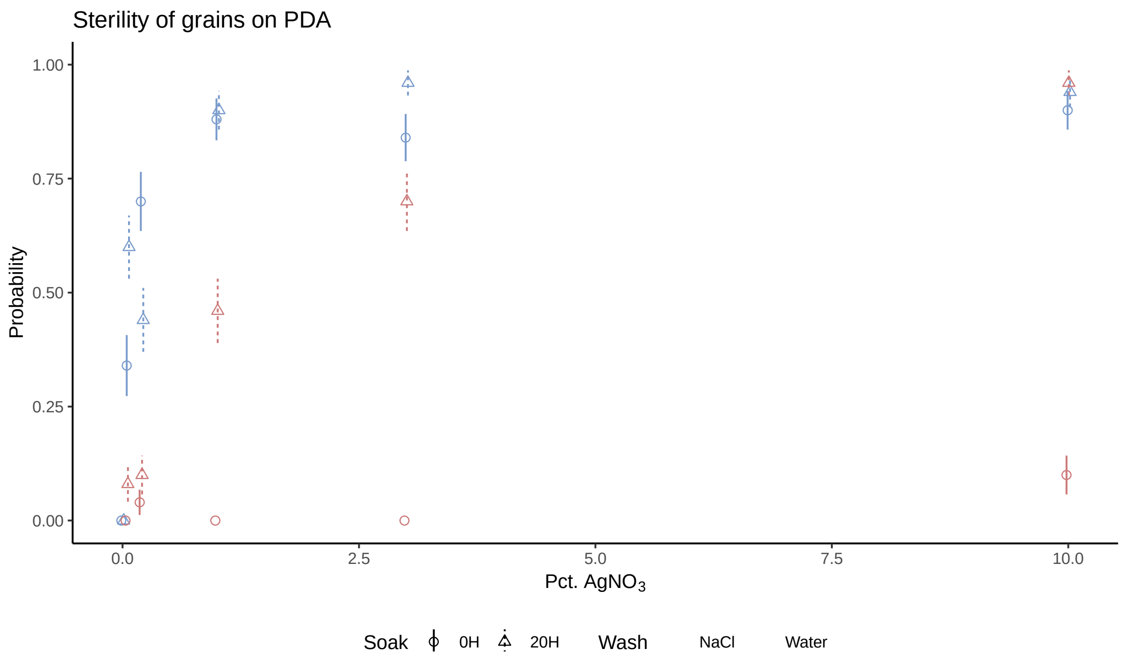

Figure S2. Sterility of grains. Probability of sterility of grains of barley after 21 days in response to concentration of $AgNO_3$. Symbols and error bars are mean probability ±SE of observed values. Lines and shaded area are regression lines with SE of the generalized linear regression model. Pre-sterilization treatment: Data in circles and solid lines (both error bars and regression lines) are from dry grains and data in triangles and dashed lines (both error bars and regression lines) are from grains soaked for 20 hours prior to sterilization. Post-sterilization treatment: Data in blue are from grains rinsed in sterile ddH_2O and data in red are from grains rinsed in 1% NaCl. The four-way interaction of [$AgNO_3$], medium, pre-sterilization treatment, and post-sterilization treatment was statistically significant: *P*-value: .002. Least-squares means and Tukey's test results are specified in detail in Table S2.

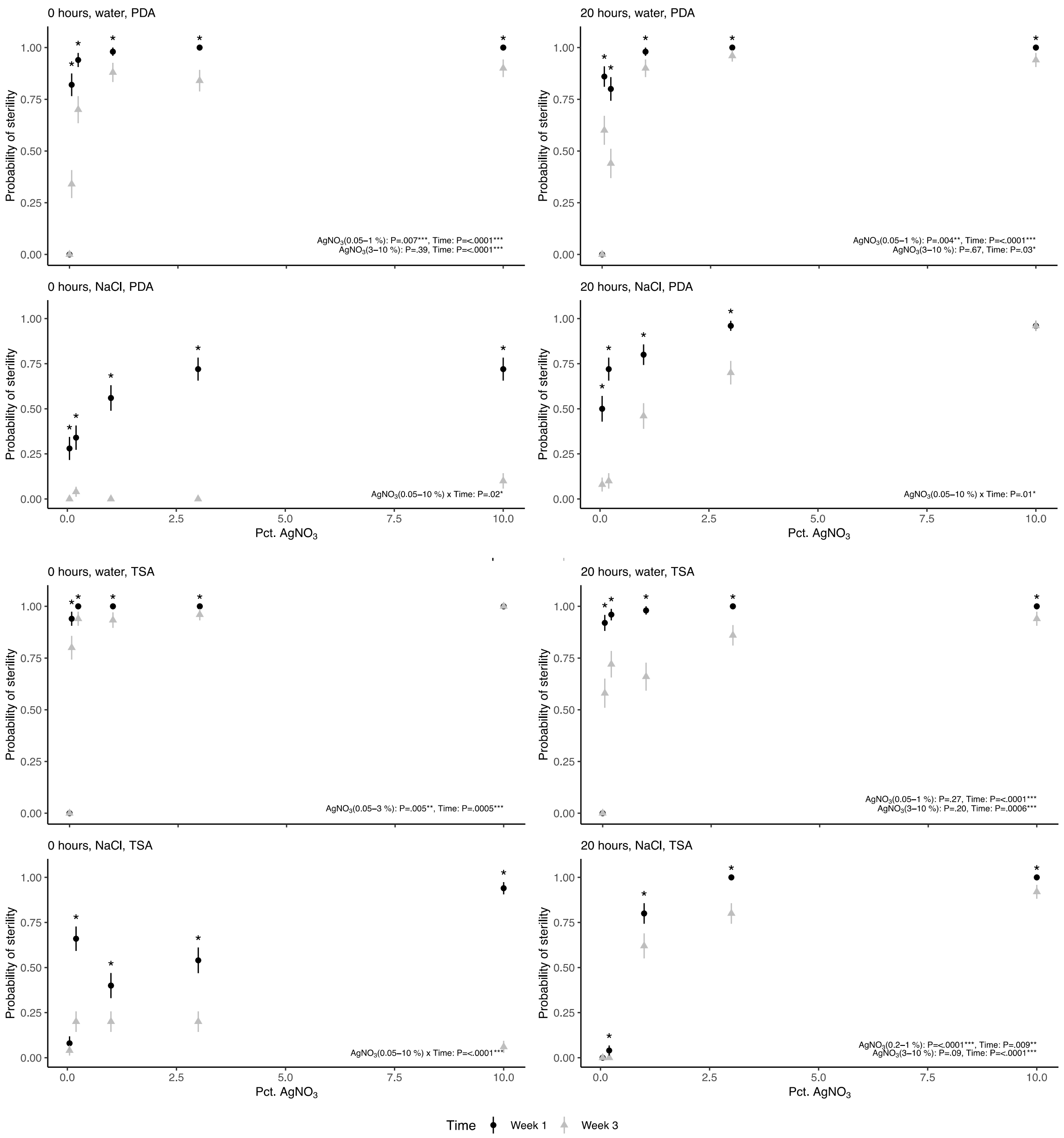

Figure S3. Difference in sterility of grains in response to concentration of $AgNO_3$ between day 7 and 21. Symbols and error bars are mean probability ±SE of observed values (no. observations = 50 except 1% $AgNO_3$, dry grains rinsed in ddH_2O on TSA plates = 45). Asterisk indicates a significant difference (p-value<0.05) between day 7 (black circles) and day 21 (gray triangles) at that particular $AgNO_3$ concentration. Means and Tukey's test results are specified in detail in Table S3.

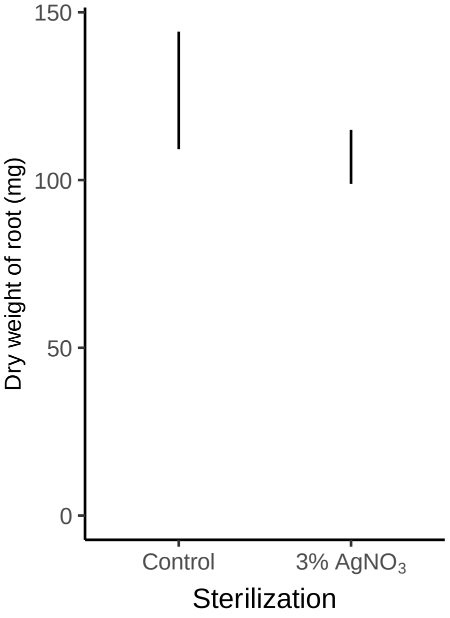

Figure S4. Root weight. Difference in root dry weight after 25 days between grains of spring barley (Hordeum vulgare L. cv. Evergreen) receiving either no sterilization (control) or 3% $AgNO_3$. n=23 for the control treatment and n=20 for the 3% $AgNO_3$ treatment. Values are given as mean (±SE). No asterisk indicates no significant difference (p-value<0.05) based on a t-test (t=-0.98, df=41, p-value=0.33).

1.2 Supplementary Tables

Table S1. Preliminary test of sterilizing agents. Mean probability of sterility (±SE) of spring barley (*Hordeum vulgare* cv. Evergreen) after sterilization with three different sterilizing agents (H_2O_2, NaOCl, $AgNO_3$) based on their recommended protocol and two controls consisting of untreated, dry grains and grains soaked in water for 7 hours. If two means share the same superscripted letter within the same response variable, then no statistically significant difference was found between those two sterilization treatments based on a Tukey's test of least-squares means adjusted for multiple comparisons (5 estimates). Figure 1 provides a visual overview of the data.

Response variable	Control: Dry grains	Control: Soaked grains	H_2O_2	NaOCl	$AgNO_3$
Sterility probability	0.00(±0.00)^{a}	0.00(±0.00)^{a}	0.00(±0.00)^{a}	0.07(±0.05)^{a}	0.83(±0.07)^{b}

Table S2. Grain germination, day 21. Back-transformed probabilities of germination (±SE) based on the generalized linear model for germination (no. observations=50 except 1% $AgNO_3$, dry grains rinsed in ddH_2O on TSA plates=45). Superscripted letters and numbers are based on a pairwise Tukey´s test (significance level of 0.05). Different superscripted letters denote a statistically significant difference between two treatment combinations of pre- and post-sterilization treatment at one fixed $AgNO_3$ concentration within a specific medium. Different superscripted numbers denote a statistically significant difference between two levels of $AgNO_3$ within one fixed combination of pre- and post-sterilization treatment. They should be compared horizontally. Figure 2 provides a visual overview of the data.

Treatment			Concentration of $AgNO_3$ % (w/w)					
Medium	Pre-	Post-	0	0.05	0.2	1	3	10
PDA	0H	ddH_2O	0.95(±0.02)^{a,1}	0.93(±0.02)^{a,1}	0.92(±0.02)^{a,1}	0.86(±0.03)^{a,1}	0.77(±0.05)^{a,2}	0.64 (±0.07)^{a,2}
		NaCl		0.93(±0.03)^{a,1}	0.93(±0.02)^{a,1}	0.93(±0.02)^{a,1}	0.92(±0.03)^{ab,1}	0.92 (±0.04)^{bc,1}
	20H	ddH_2O	0.92(±0.03)^{a,1}	0.91(±0.02)^{a,1}	0.90(±0.02)^{a,1}	0.88(±0.03)^{a,1}	0.84(±0.04)^{ab,1}	0.75 (±0.06)^{ab,1}
		NaCl		0.84(±0.04)^{a,1}	0.86(±0.03)^{a,1}	0.89(±0.03)^{a,1}	0.93(±0.03)^{b,1}	0.96 (±0.03)^{c,1}
TSA	0H	ddH_2O	0.41(±0.05)^{a,1}	0.39(±0.04)^{a,1}	0.37(±0.03)^{a,1}	0.32(±0.04)^{a,1}	0.28(±0.05)^{a,1}	0.25(±0.06)^{a,1}
		NaCl		0.53(±0.06)^{ab,1}	0.54(±0.04)^{b,1}	0.56(±0.05)^{bc,1}	0.59(±0.06)^{bc,1}	0.65(±0.07)^{b,1}
	20H	ddH_2O	0.68(±0.05)^{b,1}	0.65(±0.04)^{b,1}	0.61(±0.03)^{b,1}	0.52(±0.05)^{b,1}	0.39(±0.05)^{ab,2}	0.17(±0.05)^{a,2}
		NaCl		0.65(±0.06)^{b,1}	0.67(±0.04)^{b,1}	0.70(±0.04)^{c,1}	0.72(±0.05)^{c,1}	0.67(±0.07)^{b,1}

Table S3. Grain germination, difference between day 7 and 21. Mean probabilities of germination (±SE) (no. observations=50 except 1% $AgNO_3$, dry grains rinsed in ddH_2O on TSA plates=45). Superscripted letters are based on a pairwise Tukey´s test (significance level of 0.05). Different superscripted letters denote a statistically significant difference between week one and week three at a specific combination of pre- and post-sterilization treatment at one fixed $AgNO_3$ concentration within one medium. Figure S1 provides a visual overview of the data.

Treatment	Time	Concentration of $AgNO_3$ % (w/w)					
		0	0.05	0.2	1	3	10
0H, ddH_2O, PDA	Day 7	0.94(±0.03)^{a}	0.82(±0.05)^{a}	0.48(±0.07)^{a}	0.52(±0.07)^{a}	0.20(±0.06)^{a}	0.18(±0.05)^{a}
	Day 21	0.94(±0.03)^{a}	0.94(±0.03)^{b}	0.90(±0.04)^{b}	0.90(±0.04)^{b}	0.74(±0.06)^{b}	0.64(±0.07)^{b}
20H, ddH_2O, PDA	Day 7	0.82(±0.05)^{a}	0.96(±0.03)^{a}	0.92(±0.04)^{a}	0.58(±0.07)^{a}	0.54(±0.07)^{a}	0.18(±0.05)^{a}
	Day 21	0.86(±0.05)^{a}	0.96(±0.03)^{a}	0.92(±0.04)^{a}	0.90(±0.04)^{b}	0.80(±0.06)^{b}	0.76(±0.06)^{b}
0H, NaCl, PDA	Day 7		0.82(±0.05)^{a}	0.90(±0.04)^{a}	0.90(±0.04)^{a}	0.92(±0.04)^{a}	0.90(±0.04)^{a}
	Day 21		0.92(±0.04)^{a}	0.94(±0.03)^{a}	0.92(±0.04)^{a}	0.92(±0.04)^{a}	0.92(±0.04)^{a}
20H, NaCl, PDA	Day 7		0.84(±0.05)^{a}	0.76(±0.06)^{a}	0.92(±0.04)^{a}	0.88(±0.05)^{a}	0.86(±0.05)^{a}
	Day 21		0.86(±0.05)^{a}	0.80(±0.06)^{a}	0.94(±0.03)^{a}	0.90(±0.04)^{a}	0.96(±0.03)^{a}
0H, ddH_2O, TSA	Day 7	0.34(±0.07)^{a}	0.32(±0.07)^{a}	0.06(±0.03)^{a}	0.07(±0.04)^{a}	0.02(±0.02)^{a}	0.10(±0.04)^{a}
	Day 21	0.34(±0.07)^{a}	0.50(±0.07)^{b}	0.36(±0.07)^{b}	0.29(±0.07)^{b}	0.28(±0.06)^{b}	0.26(±0.06)^{b}
20H, ddH_2O, TSA	Day 7	0.60(±0.07)^{a}	0,44(±0.07)^{a}	0.16(±0.05)^{a}	0.18(±0.05)^{a}	0.06(±0.03)^{a}	0.04(±0.03)^{a}
	Day 21	0.62(±0.07)^{a}	0.76(±0.06)^{b}	0.56(±0.07)^{b}	0.52(±0.07)^{b}	0.38(±0.07)^{b}	0.18(±0.05)^{b}
0H, NaCl, TSA	Day 7		0.22(±0.06)^{a}	0.34(±0.07)^{a}	0.28(±0.06)^{a}	0.24(±0.06)^{a}	0.24(±0.06)^{a}
	Day 21		0.44(±0.07)^{b}	0.62(±0.07)^{b}	0.62(±0.07)^{b}	0.52(±0.07)^{b}	0.66(±0.07)^{b}
20H, NaCl, TSA	Day 7		0.60(±0.07)^{a}	0.68(±0.07)^{a}	0.38(±0.07)^{a}	0.58(±0.07)^{a}	0.48(±0.07)^{a}
	Day 21		0.66(±0.07)^{a}	0.68(±0.07)^{a}	0.64(±0.07)^{b}	0.76(±0.06)^{b}	0.66(±0.07)^{b}

Table S4. Grain sterility, day 21. Back-transformed probabilities of sterility (±SE) based on the generalized linear model for germination (no. observations=50 except 1% $AgNO_3$, dry grains rinsed in ddH_2O on TSA plates=45). At 0% $AgNO_3$, i.e. the control no grains were sterile. Data for this level is excluded from the model. Superscripted letters and numbers are based on a pairwise Tukey's test (significance level of 0.05). Different superscripted letters denote a statistically significant difference between two treatment combinations of pre- and post-sterilization treatment at one fixed $AgNO_3$ concentration within a specific medium. They should be compared vertically. Different superscripted numbers denote a statistically significant difference between two concentrations of $AgNO_3$ within one fixed combination of pre- and post-sterilization treatment. They should be compared horizontally. Figure S2 provides a visual overview of the data.

Treatment			Concentration of $AgNO_3$ % (w/w)				
Medium	Pre-	Post-	0.05	0.2	1	3	10
PDA	0H	ddH_2O	0.32(±0.10)^{a,1}	0.72(±0.07)$^{a, 2}$	0.89(±0.04)$^{a, 3}$	0.92(±0.02)$^{a, 3}$	0.91(±0.04)$^{a, 23}$
		NaCl	0.01(±0.01)^{b,1}	0.01(±4.7e^{-3})^{b,1}	0.01(±0.01)^{b,1}	0.02(±0.01)^{b,1}	0.06(±0.03)^{b,1}
	20 H	ddH_2O	0.53(±0.11)^{a,12}	0.65(±0.07)^{a,1}	0.85(±0.05)^{a,2}	0.94(±0.02)^{a,3}	0.98(±0.01)^{a,3}
		NaCl	0.05(±0.03)^{b,1}	0.10(±0.03)^{c,1}	0.38(±0.08)^{c,2}	0.77(±0.06)^{c,3}	0.97(±0.02)^{a,4}
TSA	0H	ddH_2O	0.86(±0.06)^{a,12}	0.92(±0.03)^{a,13}	0.97(±0.02)^{a,24}	0.98(±0.01)^{a,34}	0.99(±0.01)^{a,1234}
		NaCl	0.03(±0.02)^{b,1}	0.13(±0.04)^{b,1}	0.20(±0.06)^{b,1}	0.14(±0.04)^{b,1}	0.04(±0.03)^{b,1}
	20 H	ddH_2O	0.63(±0.10)^{a,123}	0.65(±0.07)^{c,1}	0.77(±0.06)^{c,24}	0.87(±0.04)^{c,35}	0.95(±0.03)^{a,45}
		NaCl	5.3e^{-6}(±1.8e^{-5})^{b,1}	0.01(±0.01)^{b,2}	0.57(±0.09)^{c,3}	0.90(±0.04)^{c,4}	0.93(±0.04)^{a,4}

Table S5. Grain sterility, difference between day 7 and 21. Mean probabilities of sterility (±SE) (no. observations=50 except 1% $AgNO_3$, dry grains rinsed in ddH_2O on TSA plates=45). Superscripted letters are based on a pairwise Tukey´s test (significance level of 0.05). Different superscripted letters denote a statistically significant difference between week one and week three at a specific combination of pre- and post-sterilization treatment at one fixed $AgNO_3$ concentration within one medium. Letters are missing when perfect separation occurs in both week one and week three rendering statistical tools obsolete. Figure S3 provides a visual overview of the data.

Treatment	Time	Concentration of $AgNO_3$ % (w/w)					
		0	0.05	0.2	1	3	10
0H, ddH_2O, PDA	Day 7	0.00(±0.00)	0.82(±0.05)^{a}	0.94(±0.03)^{a}	0.98(±0.02)^{a}	1.00(±0.00)^{a}	1.00(±0.00)^{a}
	Day 21	0.00(±0.00)	0.34(±0.07)^{b}	0.70(±0.07)^{b}	0.88(±0.05)^{b}	0.84(±0.05)^{b}	0.90(±0.04)^{b}
20H, ddH_2O, PDA	Day 7	0.00(±0.00)	0.86(±0.05)^{a}	0.80(±0.06)^{a}	0.98(±0.02)^{a}	1.00(±0.00)^{a}	1.00(±0.00)^{a}
	Day 21	0.00(±0.00)	0.60(±0.07)^{b}	0.44(±0.07)^{b}	0.90(±0.04)^{b}	0.96(±0.03)^{b}	0.94(±0.03)^{b}
0H, NaCl, PDA	Day 7		0.28(±0.06)^{a}	0.34(±0.07)^{a}	0.56(±0.07)^{a}	0.72(±0.06)^{a}	0.72(±0.06)^{a}
	Day 21		0.00(±0.00)^{b}	0.04(±0.03)^{b}	0.00(±0.00)^{b}	0.00(±0.00)^{b}	0.10(±0.04)^{b}

20H, NaCl, PDA	Day 7		0.50(±0.07)^{a}	0.72(±0.06)^{a}	0.80(±0.06)^{a}	0.96(±0.03)^{a}	0.96(±0.03)^{a}
	Day 21		0.08(±0.04)^{b}	0.10(±0.04)^{b}	0.46(±0.07)^{b}	0.70(±0.07)^{b}	0.96(±0.03)^{a}
0H, ddH$_2$O, TSA	Day 7	0.00(±0.00)	0.94(±0.03)^{a}	1.00(±0.00)^{a}	1.00(±0.00)^{a}	1.00(±0.00)^{a}	1.00(±0.00)
	Day 21	0.00(±0.00)	0.80(±0.06)^{b}	0.94(±0.03)^{b}	0.93(±0.04)^{b}	0.96(±0.03)^{b}	1.00(±0.00)
20H, ddH$_2$O, TSA	Day 7	0.00(±0.00)	0.92(±0.04)^{a}	0.96(±0.03)^{a}	0.98(±0.02)^{a}	1.00(±0.00)^{a}	1.00(±0.00)^{a}
	Day 21	0.00(±0.00)	0.58(±0.07)^{b}	0.72(±0.06)^{b}	0.66(±0.07)^{b}	0.86(±0.05)^{b}	0.94(±0.03)^{b}
0H, NaCl, TSA	Day 7		0.08(±0.04)^{a}	0.66(±0.07)^{a}	0.40(±0.07)^{a}	0.54(±0.07)^{a}	0.94(±0.03)^{a}
	Day 21		0.04(±0.07)^{a}	0.20(±0.06)^{b}	0.20(±0.06)^{b}	0.20(±0.06)^{b}	0.06(±0.03)^{b}
20H, NaCl, TSA	Day 7		0.00(±0.00)	0.04(±0.03)^{a}	0.80(±0.06)^{a}	1.00(±0.00)^{a}	1.00(±0.00)^{a}
	Day 21		0.00(±0.00)	0.00(±0.00)^{b}	0.62(±0.07)^{b}	0.80(±0.06)^{b}	0.92(±0.04)^{b}

Table S6. Sterility of germinated grains, day 21. Back-transformed probabilities of sterility of germinated grains (±SE) based on the generalized linear model for germination (no. observations for PDA=32-48 and TSA=9-38). At 0% $AgNO_3$, i.e. the control no grains were sterile. Data for this level is excluded from the model. Superscripted letters and numbers are based on a pairwise Tukey's test (significance level of 0.05). Different superscripted letters denote a statistically significant difference between two treatment combinations of pre- and post-sterilization treatment at one fixed $AgNO_3$ concentration within a specific medium. Different superscripted numbers denote a statistically significant difference between two levels of $AgNO_3$ within one fixed combination of pre- and post-sterilization treatment. They should be compared horizontally. Figure 3 provides a visual overview of the data.

Treatment			Concentration of $AgNO_3$ % (w/w)				
Medium	Pre-	Post-	0.05	0.2	1	3	10
PDA	0H	ddH$_2$O	0.28(±0.11)$^{ab, 1}$	0.77(±0.07)$^{a, 2}$	0.93(±0.03)$^{a, 3}$	0.95(±0.02)$^{a, 3}$	0.94(±0.04)$^{a, 23}$
		NaCl	0.01(±0.01)$^{c, 1}$	4.6e^{-3}(±4.0e^{-3})$^{b, 1}$	0.01(±0.01)$^{b, 1}$	0.01(±0.01)$^{b, 1}$	0.05(±0.03)$^{b, 1}$
	20 H	ddH$_2$O	0.57(±0.13)$^{a, 12}$	0.64(±0.10)$^{a, 1}$	0.87(±0.05)$^{a, 23}$	0.97(±0.02)$^{a, 4}$	1.00(±0.01)$^{a, 34}$
		NaCl	0.05(±0.03)$^{bc, 1}$	0.10(±0.04)$^{c, 1}$	0.40(±0.10)$^{c, 2}$	0.80(±0.06)$^{c, 3}$	0.98(±0.02)$^{a, 4}$
TSA	0H	ddH$_2$O	0.92(±0.06)$^{a, 1}$	0.96(±0.04)$^{a, 1}$	0.99(±0.02)$^{a, 1}$	0.99(±0.01)$^{a, 1}$	1.00(±0.01)$^{a, 1}$
		NaCl	4.3e^{-3}(±0.01)$^{b, 1}$	0.12(±0.06)$^{b, 1}$	0.25(±0.10)$^{b, 1}$	0.08(±0.04)$^{b, 1}$	3.5e^{-3}(±0.01)$^{b, 1}$
	20 H	ddH$_2$O	0.77(±0.10)$^{a, 1}$	0.89(±0.05)$^{a, 1}$	0.93(±0.04)$^{ac, 1}$	0.94(±0.03)$^{a, 1}$	0.93(±0.07)$^{a, 1}$
		NaCl	7.1e^{-7}(±3.1e^{-6})$^{b, 1}$	0.01(±0.01)$^{b, 2}$	0.74(±0.10)$^{c, 3}$	0.95(±0.03)$^{a, 4}$	0.93(±0.05)$^{a, 34}$

Table S7. Residual Ag in grains. Residual Ag (μg Ag per g grain) left on and in grains of spring barley (*Hordeum vulgare* cv. Evergreen) after sterilization with different combinations of $AgNO_3$ and pre- and post-sterilization treatment. Values are given as back-transformed least-squares means (±SE) based on three replicates. If two least-squares means shared the same superscripted letter, then no statistically significant difference was found between those two specific combinations of $AgNO_3$ concentration and pre- and post-sterilization treatments based on a Tukey's test of least-squares means adjusted for multiple comparisons (22 estimates). Figure 4 provides a visual overview of the data.

Treatment		Concentration of $AgNO_3$ % (w/w)					
Pre-	Post-	0	0.05	0.2	1	3	10
0H	ddH$_2$O	1.56(±0.47)^{a}	88.84(±26.51)^{efg}	38.83(±11.59)^{cdef}	214.70(±64.07)^{gh}	601.50(±179.51)^{h}	587.95(±175.47)^{h}
	NaCl		13.63±(4.07)^{bcd}	9.19±(2.74)^{bc}	12.63±(3.77)^{bc}	12.01±(3.58)^{bc}	13.55±(4.04)^{bcd}
20 H	ddH$_2$O	1.55(±0.46)^{a}	34.94(±10.43)^{cdef}	33.66(±10.05)^{cdef}	77.80(±23.22)^{efg}	67.53(±20.15)^{defg}	105.65(±31.53)^{fg}
	NaCl		18.35(±5.48)^{bcde}	10.04(±3.00)^{bc}	5.39(±1.61)^{ab}	10.11(±3.02)^{bc}	12.79(±3.82)^{bc}
